# Thyroid-Stimulating Hormone (TSH) Concentration at Birth in Belgian Neonates and Cognitive Development at Preschool Age

**DOI:** 10.3390/nu7115450

**Published:** 2015-11-02

**Authors:** Caroline Trumpff, Jean De Schepper, Johan Vanderfaeillie, Nathalie Vercruysse, Herman Van Oyen, Rodrigo Moreno-Reyes, Jean Tafforeau, Jean Vanderpas, Stefanie Vandevijvere

**Affiliations:** 1 Unit of Public Health and Surveillance, Scientific Institute of Public Health, Rue Juliette Wytsman 14, 1050 Brussels, Belgium; Herman.VanOyen@wiv-isp.be (H.V.O.); Jean.Tafforeau@wiv-isp.be (J.T.); Stefanie.Vandevijvere@wiv-isp.be (S.V.); 2 Department of Paediatric Endocrinology, UZ Brussel, Vrije Universiteit Brussel, Avenue du Laerbeek 101, 1090 Brussels, Belgium; Jean.DeSchepper@uzbrussel.be; 3 Faculty of Psychology and Educational Sciences, Vrije Universiteit Brussel, Boulevard de la Plaine 2, 1050 Brussels, Belgium; jvdfaeil@vub.ac.be; 4 Faculty of Psychology and Educational Sciences, Université Libre de Bruxelles, avenue F.D. Roosevelt 50, 1050 Brussels, Belgium; nvercr@ulb.ac.be; 5 Department of Nuclear Medicine, Erasmus Hospital, Université Libre de Bruxelles, Route de Lennik 808, 1070 Brussels, Belgium; rmorenor@ulb.ac.be; 6 Medical Microbiology Laboratory, Communicable and Infectious Diseases, Scientific Institute of Public Health, Rue Engeland 642, 1180 Brussels, Belgium; Jean.Vanderpas@wiv-isp.be

**Keywords:** cognitive development, thyroid-stimulating hormone, preschool children, iodine deficiency, pregnancy

## Abstract

The main objective of the study was to investigate the effect of MID during late pregnancy, assessed by the thyroid-stimulating hormone (TSH) concentration at neonatal screening, on cognitive development of preschool children. A retrospective cohort study including 311 Belgian preschool children of 4–6 years old was conducted. Children were selected at random from the total list of neonates screened in 2008, 2009, and 2010 by the Brussels new-born screening center. Infants with congenital hypothyroidism, low birth weight, and/or prematurity were excluded from the selection. The selected children were stratified by gender and TSH-range (0.45–15 mIU/L). Cognitive abilities were assessed using Wechsler Preschool and Primary Scale of Intelligence—third edition. In addition, several socioeconomic, parental, and child confounding factors were assessed. Neonatal TSH concentration—a surrogate marker for MID—was not associated with Full Scale and Performance IQ scores in children. Lower Verbal IQ scores were found in children with neonatal TSH values comprised between 10–15 mIU/L compared to lower TSH levels in univariate analysis but these results did not hold when adjusting for confounding factors. Current levels of iodine deficiency among pregnant Belgian women may not be severe enough to affect the neurodevelopment of preschool children.

## 1. Introduction

Iodine is necessary for the synthesis of the thyroid hormones thyroxin (T4) and tri-iodothyronine (T3) [1], which are essential for the development of the brain during fetal and early postnatal life. In these critical periods, severe iodine deficiency can induce irreversible brain damage in the fetus and the infant, resulting in retarded cognitive or/and psychomotor development [2].

While severe iodine deficiency is disappearing in developed countries, mild iodine deficiency (MID) in women of child-bearing age and pregnant women is still present in some European countries including Belgium [3,4,5]. There is some evidence that MID during pregnancy may be associated with impaired neurodevelopment of the offspring [6]. An eventual impact of maternal MID on neurodevelopment of the children is a major public health concern and justifies further study given the limited data available.

Thyroid-stimulating hormone (TSH) concentration in whole blood measured at birth has been proposed as an indicator of maternal iodine status during late pregnancy [7,8]. Thyroid hormone level is dependent on iodine nutrition and iodine stocks when thyroid disease is excluded [9]. Liberation of TSH occurs to stimulate production of the thyroid hormone when it is insufficient. When the concentration of thyroid hormone returns to normal, TSH liberation stops. In the case of iodine deficiency, the thyroid hormone level does not adapt. TSH continues to be secreted and its concentration within the blood increases.

Previous studies have shown an association between elevated neonatal TSH (>5 mIU/L) concentration (cord serum TSH or whole blood) and subclinical impairments of cognitive development during childhood [10,11,12]. These studies are scarce and they used very small sample sizes [10,11,12]. In addition, other pregnancy-related factors than MID may explain both the variation of neonatal TSH concentrations in blood and impairments in cognitive development such as premature birth or low birth weight [6]. Additional studies are needed to understand the potential harmful effect of mild neonatal thyroid dysfunction on later cognitive development.

The aim of this study was to assess the association between neonatal TSH concentration and cognitive development at preschool age in Belgium, a country where MID is frequent during pregnancy. This association was studied using a sample size calculation and excluding children born prematurely and/or with a low birth weight and without congenital hypothyroidism, as well as taking into account confounding factors using multivariate analysis. In addition, this is the first study to use a sample stratified by sex and TSH levels in order to study this association.

## 2. Materials and Methods

### 2.1. Study Population

We used data from a Belgian retrospective cohort study, the PsychoTSH study, including 315 children aged 4, 5, and 6 years old with a neonatal TSH concentration in the range 0.45–15 mIU/L (micro international unit/liter) [13]. A sample size of 315 children was determined based on a detection probability of 95%, a significance level alpha of 5% and a correlation factor between TSH and IQ of 0.2. An anticipated drop-out of 20% was taken into account. Children were randomly selected from the total list of neonates screened in 2008 (*N* = 29,013), 2009 (*N* = 29,602) and 2010 (*N* = 30,126) by the Brussels New-born Screening Centre for Metabolic Disorders (Laboratoire de Pédiatrie, Université Libre de Bruxelles (ULB), Brussels, Belgium). Children with congenital hypothyroidism, prematurity (<37 weeks), and low birth weight (<2500 g) were excluded from the sample frame before the selection procedure. The sampling was stratified by gender and TSH level to ensure that the whole range of neonatal TSH was represented. For each gender and TSH-interval (0.45–1, 1–2, 2–3, 3–4, 4–5, 5–6, 6–7, 7–8, 8–9 and 9–15 mIU/L) 19 new-borns were selected. Within each stratum, for each selected infant, three replacements were selected, in case of refusals or non-contactable individuals. Children with neurological diseases were excluded from the study during the recruitment process.

### 2.2. Approval by Ethical Committee for Human Subjects and the Privacy Commission

A written informed consent was obtained from the parents before the test administration. The protocol and the consent form were approved by *the Ethical Committee of the Erasmus hospital* (ULB, Brussels, Reference CCB: B40620109191) in accordance with the Code of Ethics of the World Medical Association for experiments involving humans (Declaration of Helsinki). This study was also approved by the Belgian *Commission for the Protection of Privacy* (Reference: RN 29/2012).

### 2.3. TSH Measurement

TSH levels were measured by a commercial time-resolved fluoroimmunoassay (Autodelfia method) in dried blood spots on filter paper, which were collected by heel stick between 3 to 5 days after birth [14]. The reproducibility of the TSH values in the range 0–15 mIU/L was tested. At 50 different TSH values (e.g., 0.5, 2 mIU/L, 3 mIU/L, 4 mIU/L, 6 mIU/L, 8 mIU/L, 10 mIU/L) TSH was reanalyzed twice in order to determine the coefficient of variation. It was found that for the TSH values ranging from 0.9 to 10 mIU/L the variation coefficient of was below 20%. For values below 0.9 mIU/L a TSH value of 0.45 mIU/L was set in the study.

### 2.4. Cognitive Assessment

Cognitive development was assessed by the French version of the Wechsler Preschool and Primary Scale of Intelligence—third edition (WPPSI-III) [15]. The WPPSI-III is a test administered individually which enables one to measure the intelligence of children aged between 2 years 6 months and 7 years 3 months. The test is composed of 14 subtests: seven verbal tests, five performance tests, and two processing speed tests. These subtests enable the calculation of a Full Scale Intelligence Quotient (FIQ), a Performance IQ (PIQ), a Verbal IQ (VIQ), a Processing Speed Quotient and an optional score of General Language Knowledge. In this study, only the methodology for the age group 4 years to 7 years 3 months was used and only 7 core subtests were administered: Block Design, Information, Matrix Reasoning, Vocabulary, Picture Concepts, Word Reasoning and Coding. Cognitive testing was undertaken by psychologists who were blinded for the neonatal TSH levels of the selected children. Test administration was performed during a home visit. The parents were asked to provide a quiet room and leave the child alone with the psychologist in order to avoid distraction.

### 2.5. Data Collection of Descriptive Variables, Effect Modifiers and Covariates

The body weight, height, and head circumference of the child were measured during the home visit. A sample of urine was collected from the child during the home visit and analyzed using a modification of the Sandell-Kolthoff reaction with spectrophotometric detection [16] at the Clinical Chemistry Laboratory of the Erasmus Hospital (ULB, Brussels).

Date of birth, date of blood sampling, pregnancy duration and body weight at birth were provided by the Brussels new-born screening center for metabolic disorders (Laboratoire de Pédiatrie, ULB, Brussels).

Data concerning body length at birth, head circumference at birth, and Apgar score was collected from the health booklet of the child during the home visit.

Additional information was collected using a self-administered questionnaire filled in by the mother: pre-pregnancy body mass index (BMI) of the mother, thyroid disease, drug intake, chronic disease, alcohol consumption, smoking habits, weight gain and intake of nutritional supplements of the mother during pregnancy, maternal age at birth, reproductive history, parity, gravidity, type of delivery, perinatal asphyxia, health condition of the new-born, child’s negative life events, maternal mental health, maternal social support, marital discord, and parent-child interactions. Maternal mental health was assessed using the General Health Questionnaire—12 [17] items and vitality scale questionnaire of the Short Form Health Survey—36 [18].

### 2.6. Data Analysis

Statistical analysis was performed using SAS statistical software 9.3 (SAS Institute Inc., Cary, NC, USA) for univariate analysis and Stata version 13 (StataCorp, College Station, TX, USA) for multivariate analysis. Statistical tests were two-sided and tests with *p*-value <0.05 were considered statistically significant. The missing values were kept as missing and the analyses were restricted to subjects with complete data on the variables involved in the analysis. Neonatal TSH values and UIE were presented as median and IQR and IQ scores were presented as mean and SD. Neonatal TSH values were classified in three groups: below 5 mIU/L, between 5 and 9 and equal or above than 10 mIU/L.

Student *t*-test, anova with Bonferroni correction and simple linear regression were performed to examine univariate associations between IQ and studied parameters. Association between TSH level and IQ was assessed using Pearson correlation.

Multivariate linear and logistic regression models were used to study the predictors of variation of IQ scores in children. All continuous variables and categorical variables associated with IQ scores with a test *p* < 0.20 were included in the selection procedure [19]. A multiple linear regression model was built, a stepwise backward selection procedure with an entry probability of 0.10 and exit probability of 0.15 was used to define variables to be inserted into the model. Univariate association between these variables were tested with Pearson correlation. Highly correlated variables were not included together in the model. Normal plot of residuals was used to check the normality of the residuals distribution. Linearity and homoscedasticity of residuals were checked by examination of the plot of standardized residuals. Colinearity between predictors was tested using the test of variance inflation factor (VIF) and individuals’ VIF for each parameter in the model were around 1.

Logistic regression was used to estimate the risk of having a developmental score below 85 (one standard deviation below the mean of 100). A stepwise selection with an exit probability of 0.15 was used to determine the variable to insert into the final logistic regression model. For the logistic regression, adjusted odds ratios were used and 95% CI was derived from the final model. The adequacy of regression models has been tested by the Hosmer and Lemeshow test.

## 3. Results

### 3.1. Demographical Characteristics, Neonatal Thyroid Status and Current Iodine Status of the Study Sample

Descriptive and demographic characteristics of the study population according to gender are shown in Table 1. In total, 311 children (*N* = 137 girls) aged 4, 5, and 6 years were included in the study. The median (range) TSH level of the study sample was 3.6 mIU/L (1.8–5.8 (IQR), 0.45–13.9 (min-max)). The median iodine concentration of the sample was 138.8 µg/L (87.7–238.7 (IQR)) indicating iodine sufficiency of the study population.

**Table 1:** Descriptive and demographics characteristics of the study population according to gender. PsychoTSH study, Belgium, 2008–2014.

TSH Levels (mIU/L)	Total		Boys		Girls	
	*N*	%	*N*	%	*N*	%
0.45–1	45	14.5	25	8	20	6.4
2	36	11.6	22	7.1	14	4.5
3	40	12.9	22	7.1	18	5.8
4	40	12.9	20	6.4	20	6.4
5	37	11.9	21	6.8	16	5.1
6	39	12.5	20	6.4	19	6.1
7	35	11.3	21	6.8	14	4.5
8	14	4.5	10	3.2	4	1.3
9	14	4.5	6	1.9	8	2.6
10–15	11	3.5	7	2.3	4	1.3
**Age at examination**						
4 year	234	75.5	131	42.2	104	33.3
5 year	69	22.3	39	12.6	30	9.6
6 year	7	2.2	4	1.2	3	1
**Maternity hospital**						
Brussels	205	65.9	127	40.8	78	25.1
Wallonia	103	33.1	46	14.8	57	18.3
Flanders	3	1	1	0.3	2	0.6
**Children’s ethnicity**						
Europe (Caucasian)	250	84.5	132	48	106	38.6
Asia	3	1	2	0.7	1	0.4
Sub-Saharan Africa	15	5.1	5	1.8	6	2.2
North Africa	28	9.5	13	4.7	9	3.3
**Mother’s ethnicity**						
Europe (Caucasian)	246	82.3	141	47.2	105	35.1
Asia	5	1.7	2	0.7	3	1
Sub-Saharan Africa	19	6.4	9	3	10	3.3
North Africa	29	9.7	18	6	11	3.7
**Delivery**						
Normal	232	75.82	130	42.48	102	33.33
Caesarian section	43	14.05	23	7.52	20	6.54
Vacuum extraction	31	10.13	18	5.88	13	4.25
**Apgar score at 5 min**						
10	195	77.69	102	40.64	93	37.05
9	39	15.54	21	8.37	18	7.17
8	12	4.78	9	3.59	3	1.2
<8	5	1.99	3	1.2	2	0.8

Abbreviation: *N*, Number of subjects.

### 3.2. Results of Cognitive Assessment

The mean FIQ was 96.7 (16.4 (SD), 42–142 (min-max)), the mean PIQ was 98.4 (15.7 (SD), 46–142 (min-max)) and the mean VIQ was 96.8 (17.9 (SD), 50–139 (min-max)). In univariate analysis, several children, maternal, and socio-economics characteristics were associated with IQ scores (see Table 2 and Table 3).

Univariate associations between cognitive scores at preschool age and iodine status are shown in Table 4.

No differences were found in FIQ and PIQ scores in children with TSH levels lower than 5 mIU/L compared to TSH levels between 5 and 9 mIU/L and between 10 and 15 mIUL/L. Lower VIQ scores were found in children with TSH levels between 10–15 mIU/L compared to lower levels (0.45–9 mIU/L) (*p* = 0.006). After correction for confounding factors—such as monthly income of the household, education level of the mother or child bilingualism—by multiple regression analysis (see below), this difference was no longer significant.

The use of iodized salt was related to higher PIQ scores (*p* = 0.013) and the intake of dietary supplement by the children was associated with higher FIQ (*p* = 0.023) and PIQ (*p* = 0.020).

No associations were found between cognitive scores at preschool age and the following factors: Apgar score at 5 min, health problems at birth, neonatal hospital attendance, previous cognitive assessment, school attendance, child’s negative life events, child custody, rural or urban residency, delivery type, previous miscarriage, Graves’ disease or Hashimoto’s thyroiditis during pregnancy, hypothyroidism during pregnancy, diabetes during pregnancy (data not shown).

**Table 2:** Association of cognitive scores of the Wechsler Preschool and Primary Scale of Intelligence at preschool age with infant, maternal, and household characteristics: categorical variables. PsychoTSH study, Belgium, 2008–2014.

	*N*	%	FIQ			PIQ			VIQ		
			Mean	SD	*P* ^a^	Mean	SD	*P* ^a^	Mean	SD	*P* ^a^
**Infant and children characteristics**											
**Gender**					**0.045**			0.852			0.406
Male	174	56	95.1	16.5		98.2	15.0		95.7	18.6	
Female	137	44	98.9	16.5		98.5	16.2		97.5	18.4	
**Breastfeeding at 6 months**					0.375			0.296			0.449
<6 months	150	48.5	96.2	16.3		97.5	16.0		95.5	19.1	
>6 months	123	39.8	98.3	16.8		100.0	14.8		98.1	19.1	
No breastfeeding	36	11.7	94.4	17.0		96.2	15.7		95.0	15.1	
**Language**					**0.035**			0.792			**0.0004**
French	239	77.1	97.9	16.1		98.2	15.5		98.5	17.3	
French and other	71	22.9	93.1	17.6		98.8	15.6		89.5	21.4	
**Maternal characteristics**											
**Parity—First child**					0.157			**0.044**			0.062
Yes	132	42.7	98.3	17.0		100.4	16.0		98.8	18.3	
No	177	57.3	95.6	16.2		96.8	15.0		94.7	18.8	
**Smoking during pregnancy**					0.081			0.503			0.059
≥10 cigarettes/day	7	2.3	86.0	15.8		94.4	12.6		83.4	14.0	
<10 cigarettes/day or no smoking	304	97.7	97.1	16.5		98.4	15.6		96.8	18.5	
**Alcohol during pregnancy**					0.118			0.055			0.427
Non consumer	209	68.1	96.0	16.6		97.4	16.1		95.9	18.7	
Still consuming	98	31.9	99.1	16.2		101.0	13.1		97.7	18.6	
**Maternal characteristics**											
**Maternal social support ^b^**					**0.0009**			**0.003**			**0.024**
3 or less	98	31.7	92.4	17.6		94.5	17.2		93.2	19.1	
4 or more	211	68.3	99.1	15.6		100.1	14.3		98.3	17.9	
**Score of psychological distress**					0.635			0.891			0.511
<4	259	88.1	96.7	15.9		98.4	14.9		96.7	18.1	
>4	35	11.9	98.1	21.1		98.8	18.6		94.5	23.1	
**Vitality Index**					0.067			0.074			0.105
Optimal vitality	269	89.1	97.5	16.5		98.8	15.4		97.3	18.2	
Sub-optimal vitality	33	10.9	91.9	16.4		93.8	15.0		91.8	21.3	
**Socio-economics characteristics**											
**Monthly incomes**					**<0.0001**			**0.013**			**<0.0001**
<2000 euros	38	12.7	87.1	17.2		92.6	17.9		85.8	19.4	
≥2000 euros	262	87.3	98.4	15.9		99.3	15.0		98.3	17.8	
**Mother’s education level**					<0.0001 *			0.283			<0.0001 *
No/primary	10	3.3	84.2	14.7		93.3	10.8		80.4	21.7	
Lower high school	22	7.2	93.0	18.4		101.0	18.5		89.2	17.4	
Upper high school	50	16.4	89.3	17.3		95.3	15.7		87.2	19.8	
Graduate school or university	223	73.1	99.4	15.6		98.9	15.4		99.9	17.1	

Abbreviations: FIQ, Full Scale Intelligence Quotient; *N*, Number of subjects; *P*, *p*-value PIQ, Performance Intelligence Quotient; SD, Standard Deviation; VIQ, Verbal Intelligence Quotient; ^a^ *p*-value from simple *t*-test or Anova; ^b^ Number of people to call in case of problems; * Significant with Bonferroni correction for multiple testing.

**Table 3:** Association of cognitive scores at preschool age with infant, maternal, and household characteristics: continuous variables. PsychoTSH study, Belgium, 2008–2014.

				**Pearson Correlation**					
				**FIQ**		**PIQ**		**VIQ**	
	***N***	**Median**	**IQR**	***R***	***P***	***R***	***P***	***R***	***P***
**TSH, mIU/L**	311	3.6	1.8–5.8	0.5	0.396	0.1	0.687	0.1	0.112
				**Univariate Linear Regression**					
	***N***	**Mean**	**SD**	***b* (SE)**	***P***	***b* (SE)**	***P***	***b* (SE)**	***P***
**Term pregnancy (weeks)**	308	39.3	1.6	0.1 (0.6)	0.913	0.1 (0.6)	0.891	0.1 (0.7)	0.861
**Birth weight (g)**	311	3394.2	429.8	0.0 (0.0)	0.820	0.0 (0.0)	0.599	0.0 (0.0)	0.377
**Birth length (cm)**	310	50	3.2	−0.2 (0.3)	0.540	0.3 (0.3)	0.240	−0.4 (0.3)	0.240
**Birth HC (cm)**	269	34.6	3.1	−0.1 (−0.2)	0.820	0.0 (0.3)	0.890	−0.2 (0.4)	0.560
**Actual age (years)**	310	4.7	0.5	0.7 (1.9)	0.736	−1.4 (1.8)	0.447	1.5 (2.2)	0.481
**Actual weight (kg)**	309	18.8	5.5	−0.1 (0.2)	0.484	−0.1 (0.2)	0.420	−0.1 (0.2)	0.522
**Actual height (cm)**	309	107.2	11	0.1 (0.1)	0.514	0.0 (0.1)	0.570	0.1 (0.1)	0.377
**Actual HC (cm)**	308	51.2	3.2	0.1 (0.3)	0.828	0.0 (0.3)	0.928	0.1 (0.3)	0.793
**Mother age (years)**	310	36.5	5.1	0.3 (0.2)	0.114	0.1 (0.2)	0.401	0.4 (0.2)	0.089
**Mother weight (kg)**	307	65.3	11.9	0.0 (0.1)	0.749	0.0 (0.1)	0.682	−0.1 (0.1)	0.179
**Mother height (cm)**	305	165.8	6	0.2 (0.2)	0.251	0.2 (0.1)	0.181	0.2 (0.2)	0.381
**Weight gain during pregnancy (kg)**	288	13.4	5.9	−0.2 (0.2)	0.297	−0.2 (0.2)	0.164	−0.3 (0.2)	0.134
**Nb of cigarettes during pregnancy**	18	8.3	6.8	−1.0 (0.5)	**0.040**	−0.6 (0.5)	0.280	−0.9 (0.4)	**0.030**
**Nb of people in the household**	301	3.3	1.1	−0.2 (0.9)	0.839	0.4 (0.8)	0.630	−0.2 (1.0)	0.825

Abbreviations: FIQ, Full Scale Intelligence Quotient; *N*, Number of subjects; *P*, *p*-value PIQ, Performance Intelligence Quotient; SD, Standard Deviation; SE, Standard Error; VIQ, Verbal Intelligence Quotient.

**Table 4:** Cognitive scores at preschool age and markers of iodine status. PsychoTSH study, Belgium, 2008–2014.

	*N*	%	FIQ			PIQ			VIQ		
			Mean	SD	*P* ^a^	Mean	SD	*P* ^a^	Mean	SD	*P* ^a^
**Pregnancy and neonatal period**											
**Neonatal TSH level, mIU/L**					0.058			0.533			0.006 *
<5	198	63.7	95.9	16.9		98.0	16.3		94.9	19.8	
5–9	102	32.8	99.4	14.2		99.4	13.1		100.8	14.2	
≥10	11	3.5	88.9	26.1		94.5	21.6		86.7	22.8	
**Vitamins during pregnancy**					0.314			0.254			0.239
Containing iodine	51	43.6	99.5	14.5		100.6	12.6		98.1	17.4	
No vitamins	66	56.4	96.4	17.0		97.4	16.5		93.9	20.1	
**Children characteristics**											
**Urinary iodine concentration, µg/L**					0.268			0.555			0.349
<100	81	30.4	99.7	16.2		99.3	14.1		99.1	20.2	
100–149	66	24.8	95.2	16.5		96.5	16.8		94.5	20.0	
150–294	60	22.6	97.5	17.8		100.1	15.2		96.9	17.5	
≥250	59	22.2	94.9	15.4		99.2	15.5		94.0	16.4	
**Household salt**					0.093			**0.013**			0.158
Iodized salt	96	34.5	98.8	16.1		101.3	14.0		98.4	18.3	
Non iodized salt	182	65.5	95.3	16.7		96.4	16.0		95.1	18.6	
**Addition of salt during meal**					0.343			0.613			0.236
Always	9	2.9	91.6	21.1		96.6	20.7		87.6	22.3	
Sometimes	50	16.3	99.3	15.4		100.3	12.8		99.1	18.6	
Never	248	80.8	96.4	16.3		98.1	15.6		96.6	17.8	
**Child dietary supplement intake**					**0.023**			**0.020**			0.107
Yes	196	65.6	98.4	16.7		99.8	15.7		97.7	18.5	
No	103	34.4	93.8	16.6		95.4	15.0		94.0	19.1	
**Milk consumption**					0.940			0.787			0.961
Less than 100 g	118	41.3	96.8	16.0		97.9	16.5		96.7	17.4	
About 100 g	124	43.4	97.1	16.2		98.6	13.4		96.3	20.4	
More than 100 g	36	12.6	97.9	19.9		100.8	16.5		97.8	19.1	
No milk	8	2.8	93.9	20.2		97.1	15.0		94.1	23.0	
**Fish consumption**					0.784			0.210			0.426
Less than 100 g	130	46.3	97.0	17.4		98.5	15.9		96.7	19.6	
About 100 g	116	41.3	96.4	16.8		97.0	15.8		97.0	17.9	
More than 100 g	27	9.6	99.7	13.3		103.0	10.6		97.5	19.4	
No fish	8	2.8	94.0	11.1		104.4	11.3		85.6	20.7	
**Child food allergy**					0.918			0.760			0.649
Yes	26	8.6	96.5	15.9		97.3	13.1		98.2	18.4	
No	277	91.4	96.9	16.8		98.3	15.9		96.4	18.7	

Abbreviations: FIQ, Full Scale Intelligence Quotient; PIQ, Performance Intelligence Quotient; VIQ, Verbal Intelligence Quotient; ^a^ *p*-value from simple *t*-test or Anova; * Significant with Bonferroni correction for multiple testing.

### 3.3. Predictors of IQ Scores Assessed by Multiple Linear Regression Analysis

The results of multiple linear regressions of factors explaining variation of IQ scores at preschool age are shown in Table 5. TSH concentration at birth was not associated with any cognitive scores. The predictors of higher IQ scores were higher household income (FIQ, *p* = 0.007; PIQ, *p* = 0.014; VIQ, *p* = 0.022), higher mother’s education level (FIQ, *p* = 0.017; PIQ, *p* = 0.046; VIQ, *p* ≤ 0.0001), and mother’s optimal vitality (FIQ, *p* = 0.04, PIQ, *p* = 0.013 VIQ, *p* = 0.012). Female gender was associated with higher FIQ (*p* = 0.019). A positive association was found between PIQ and child dietary supplement intake (*p* = 0.037), use of iodized household salt (*p* = 0.007), and stronger maternal social support (*p* = 0.003). Bilingualism was associated with lower VIQ (*p* = 0.001) and being the first born was associated with higher VIQ (*p* = 0.012).

**Table 5:** Multiple linear regression of factors explaining variation cognitive scores at preschool age. PsychoTSH study, Belgium, 2008–2014.

	FIQ (*N* = 274)	*P*	PIQ (*N* = 243)	*P*	VIQ (*N* = 243)	*P*
	*R*^2^ = 13%		*R*^2^ = 12%		*R*^2^ = 12%	
	*b* (SE)		*b* (SE)		*b* (SE)	
**Gender**		**0.019**		¶		¶
Male	Ref					
Female	4.53 (1.92)					
**Monthly income**		**0.007**		**0.014**		**0.022**
≥2000 euros	Ref		Ref		Ref	
<2000 euros	−9.03 (3.32)		−8.14 (3.28)		−7.86 (3.42)	
**Mother’s education level**		**0.017**		**0.046**		**<0.0001**
Graduate school or university	Ref		Ref		Ref	
Lower	−5.87 (2.44)		5.14(2.56)		−10.84 (2.53)	
**Child dietary supplement intake**		**0.017**		**0.037**		¶
Yes	Ref		Ref			
No	−4.88 (2.03)		−4.30 (2.04)			
**Vitality Index**		**0.040**		**0.013**		**0.012**
Optimal vitality	Ref		Ref		Ref	
Sub-optimal vitality	−6.44 (3.03)		−7.89 (3.14)		−8.43 (3.34)	
**Household salt**		¶		**0.007**		¶
Iodized salt			Ref			
Non iodized salt			−5.48 (2.01)			
**Home language**		¶		¶		**0.003**
French					Ref	
French and other					−7.58 (2.53)	
**School language**		¶		¶		**0.001**
French					Ref	
Dutch					−9.84 (2.96)	
**Mother’s social support ^a^**		¶		**0.003**		¶
3 or less			Ref			
4 or more			6.48 (2.16)			
**Parity—First child**		¶		¶		**0.012**
Yes					Ref	
No					−5.21 (2.05)	

Abbreviations: FIQ, Full Scale Intelligence Quotient; PIQ, Performance Intelligence Quotient; VIQ, Verbal Intelligence Quotient; ¶ Variable not inserted in the model; ^a^ Number of persons who can be called upon in case of problems.

### 3.4. Predictors of IQ Scores Assessed by Logistic Regression Analysis

The predictors of IQ scores lower than 85 are shown in Table 6. TSH level at birth was not associated with cognitive outcomes in multiple logistic regression analysis. Predictors of IQ scores lower than 85 were lower household income (FIQ, OR, 4.23, 95% CI, 1.94–9.20, PIQ, OR, 3.99, 95% CI, 1.3–12.18), mother’s sub-optimal vitality (PIQ, OR, 2.96, 95% CI, 1.18–7.47; VIQ, OR, 3.15, 95% CI, 1.32–7.49), male gender (FIQ, OR, 1.89, 95% CI, 1.01–3.51), child bilingualism (FIQ, OR, 1.95 , 95% CI, 1.94–9.20), use of non-iodized household salt (PIQ, OR, 2.47, 95% CI, 1.1–2.57 ), and mother’s education level lower than university or graduate school (VIQ, OR, 4.74, 95% CI, 2.54–8.83). Some variables which were included in multiple linear regression analysis such as mother’s social support, school language, and parity were no longer included in the multivariate logistic regression model.

**Table 6:** Predictors of cognitive scores <85 assessed with multiple logistic regression. PsychoTSH study, Belgium, 2008–2014.

	FIQ (*N* = 297)	*P*	PIQ (*N* = 239)	*P*	VIQ (*N* = 239)	*P*
	OR (CI 95%)		OR (CI 95%)		OR (CI 95%)	
**Gender**		**0.045**		¶		0.071
Female	1				1	
Male	1.89 (1.01–3.51)				1.76 (0.95–3.25)	
**Monthly income**		**<0.0001**		**0.015**		¶
≥2000 euros	1		1			
<2000 euros	4.23 (1.94–9.20)		3.99 (1.3–12.18)			
**Mother‘s education level**		¶		0.118		**<0.0001**
Graduate school or university			1		1	
Lower			0.45 (0.16–1.22)		4.74 (2.54–8.83)	
**Child dietary supplement intake**		¶		0.132		¶
Yes			1			
No			1.73 (0.85–3.54)			
**Vitality Index**		¶				**0.010**
Optimal vitality			1	**0.021**	1	
Sub-optimal vitality			2.96 (1.18–7.47)		3.15 (1.32–7.49)	
**Household salt**		¶		**0.028**		¶
Iodized salt			1			
Non iodized salt			2.47 (1.1–2.57)			
**Home language**		**0.045**		¶		¶
French	1					
French and other	1.95 (1.01–3.78)					
**Parity—First child**		0.058		¶		0.088
Yes	1				1	
No	1.85 (0.97–3.5)				1.71 (0.92–3.19)	
**Pregnancy duration (weeks)**		¶	0.83 (0.66–1.03)	0.099		¶

Abbreviations: CI, Confidence Interval; FIQ, Full Scale Intelligence Quotient; OR, Odds ratio; PIQ, Performance Intelligence Quotient; VIQ, Verbal Intelligence Quotient; ¶ Variable not inserted in the model.

## 4. Discussion

The main objective of the present study was to investigate the association between neonatal TSH concentration and cognitive development at preschool age in Belgium, where MID during pregnancy is frequent, taking into account confounding factors. Children with a neonatal TSH above 5 mIU/L did not have a higher risk of an impaired cognition compared to those above this cut-off point. Major factors influencing IQ status in preschool children were gender, parity, child dietary supplement intake, use of iodized salt within the household, household income, mother’s education level, mother’s social support, mother’s vitality, and child bilingualism.

Data on the association between neonatal TSH concentration, either measured in cord blood or between the 3rd and 5th day of life, and cognitive development [10,11,12] are limited and contradictory. An Italian case-controlled study has found lower Full Scale and Performance cognitive scores in a small cohort (*N* = 18) of 6 to 9 year old children with transient congenital hypothyroidism or hyperthyrotrophinaemia measured in cord blood at birth (*N* = 9) in comparison with control group (*N* = 9) [10]. In Spain, a retrospective cohort study (*N* = 61) showed that children aged 3 years with an elevated TSH level measured 3 days after birth (between 5–10 mIU/L) had lower scores in Perceptual Performance Skills, Memory Development, and General Cognitive Index (*N* = 13) than those with TSH level below 5 mIU/L (*N* = 48) [11]. These two studies were performed with a rather small sample in which the confounding factors were not assessed with statistical analysis. A more recent Spanish retrospective cohort study which evaluated 178 boys aged 3 years old born between 2000–2002, recruited from a general population, found that the TSH in cord blood (TSH range 0.24–17 mIU/L) was significantly negatively correlated with general cognitive scores and quantitative scores [12]. This study, although including a larger sample size and assessing confounding factors, still needs to be criticized because it only presented results for boys without explaining the rationale behind this.

Our study corroborates with another study conducted in Scotland reporting no evidence for an association between TSH cord blood level and child neurodevelopmental scores at 5 years old (*N* = 97) [20]. Another study including 500 children found no association between variation in newborn T4 concentration and cognitive scores at 6 months and 3 years old [21].

In the present study, the sampling was done according to a sample size calculation, stratified by TSH levels and by gender to ensure that the whole range of TSH values were included in the study. In addition, cognitive outcomes of the children were tested by blinded psychologists using a validated test and before entering primary school, thereby reducing the time between TSH measurement and cognitive testing. However, the use value of neonatal TSH as a parameter for MID during late pregnancy has recently been questioned. Indeed, the actual cut-off value of 5 mIU/L was not sensitive enough to detect MID in several studies [22,23,24]. On the other hand, several studies have investigated the effect of MID during pregnancy using maternal urinary iodine excretion (UIE) concentration as an indicator. A UK study showed that lower UIE is related to a higher risk of impaired verbal abilities [25]. The Dutch Generation R Study found an association between low urinary iodine during pregnancy and impaired executive functioning in children aged 4 years old [26]. In contrast, the Dutch Generation R study, including 1525 children aged 6 years old, found no relationship between maternal low urinary iodine concentrations before 18 weeks of pregnancy and cognitive scores [27].

Future studies aiming to assess the effect of MID during pregnancy on child neurodevelopment should adopt a prospective design and use a more reliable indicator of maternal iodine deficiency during pregnancy than elevated TSH at birth; interesting alternatives could be maternal thyroid parameter or UIE from the first trimester of pregnancy. Indeed, neonatal TSH only indicates iodine status during late pregnancy and many studies showed an impact of MID on child cognitive development when appearing before the first half of pregnancy (12 or 18 weeks) [28,29,30,31,32], before the fetal thyroid gland reaches its functional maturity, and at a time when the fetus is still entirely dependent on the thyroid hormones provided by the mother.

Importantly, more studies are needed to understand the potential beneficial/harmful effect of iodine supplementation for iodine deficient pregnant women on child neurodevelopment. Indeed, relatively little is known about the positive effect of iodine supplementation in the area of MID, only two studies have found a positive effect of potassium iodine intake during pregnancy on children’s cognitive [33] and psychomotor [34] development while one study showed a deleterious effect of iodine supplementation on psychomotor development [35]. Finally, a recent randomized controlled trial found no beneficial effect of levothyroxine supplementation for pregnant women on cognitive development of the 3 year old offspring [36].

The actual cut-off used in Belgium by newborn screening for congenital hypothyroidism is 15 mU/L. The benefits of lowering this cut-off for preventing future intellectual disabilities of children are controversial [37]. Since we did not find a significant association between the highest TSH levels groups and lower IQ scores when adjusted for covariates, our results do not support a lowering of the actual TSH cut-off used in Belgium.

In our study, we found that iodized salt consumption has a protective effect on IQ performance scores of children thereby showing the importance of adequate nutritional iodine intake for child neurodevelopment. Our study showed that a various range of factors influence IQ scores such as maternal education, household income, maternal mental health, and social support or child bilingualism. These findings emphasize the importance of taking potential confounding factors into account when trying to assess the role of perinatal factors on later cognitive development because the latter may play a bigger role than the former.

The study limitations include that we used a retrospective design and the fact that even if our sample size was large with respect to one of the previous studies, we calculated that for a sample size of 311, with an error alpha of 0.05 and a power of 0.95, the minimum IQ score difference that we were able to detect was a difference of 4.3 points. Another limitation is that we studied a highly educated population (73% of the mothers had a university degree) that could have biased the results. Additionally, we could have also measured iodine status and thyroid newborn T4 and T3 to obtain all the information about newborn thyroid functioning.

The lack of association between neonatal TSH within the range of 0 to 15 mIU/L, used as a surrogate marker for MID during pregnancy, and cognitive impairment at preschool age suggests that the current level of iodine deficiency in Belgium is probably not severe enough to affect the neurodevelopment of children.

## Abbreviations List

CI	Confidence Interval
CH	Congenital Hypothyroidism
FIQ	Full Scale Intelligence Quotient
IQ	Intelligence Quotient
MID	Mild Iodine Deficiency
mIU/L	Micro International Unit/Liter
N	Number of subjects
OR	Odd Ratio
PIQ	Performance Intelligence Quotient
SD	Standard Deviation
SE	Standard Error
T3	Tri-iodothyronine
T4	Thyroxin
TH	Thyroid Hormone
TSH	Thyroid-Stimulating Hormone
ULB	Université Libre de Bruxelles
UIE	Urinary Iodine Excretion
VIQ	Verbal Intelligence Quotient
WHO	World Health Organization
WPPSI-III	Wechsler Preschool and Primary Scale of Intelligence—third edition
